# EBV-positive small cell neuroendocrine carcinoma of nasopharynx as a probably unique subtype of neuroendocrine carcinoma: a clinicopathologic study of three cases and literature review

**DOI:** 10.1186/s13000-024-01526-w

**Published:** 2024-07-24

**Authors:** Ying Chen, Ning Zhou, Caijun Huang, Xin He, Xiaodong Wang, Hao Tang, Wenyan Wang, Jiashuang Wang, Tao Li, Deyu Guo

**Affiliations:** 1 Departments of Pathology, Guiqian International General Hospital, Guiyang, Guizhou Province, China; 2 Departments of Pathology, Sichuan Province, Sichuan Mianyang 404 Hospital, Mianyang, China; 3 Departments of Imaging, Guiqian International General Hospital, Guiyang, Guizhou Province, China

**Keywords:** Epstein-Barr virus, Small cell neuroendocrine carcinoma, Nasopharynx, Clinicopathology, Biology

## Abstract

**Background:**

There is currently scarcity of information on small cell neuroendocrine carcinoma of the nasopharynx (SCNEC-nasopharynx). It is believed that this type of cancer is not associated with Epstein-Barr virus (EBV) infection and is indistinguishable from classic SCNEC occurring in other organs.

**Materials and methods:**

Herein we provided 3 cases of nasopharyngeal mass in our hospital, two males and one female. On admission, these patients were considered nasopharyngeal carcinoma with lymph node metastasis, and one of them had liver metastasis. The nasopharyngeal mucosal tissues were biopsied for pathological examination including immunohistochemistry and in situ hybridization. PubMed database was searched for articles about SCNEC-nasopharynx published up to April 2024 in any language.

**Result:**

The 3 cases had similar histological features of SCNEC in other organs but differed in rich- tumor-infiltrating lymphocytes (TILs). All of them stained for pancytokeratin (panCK) and epidermal growth factor receptor (EGFR). Case 1 and Case 2 diffusely expressed insulinoma-associated protein 1(INSM-1) and synaptophysin (Syn), Case 3 strongly stained for CD56 and Syn. Immunostaining of all 3 cases for p40, p63, TTF-1, CK20, S-100 and NUT showed negative. BRG-1, INI-1 and Rb were retained. And p53 all showed wild-type expression. The Ki-67 labeling indiced of case 1, 2, and 3 were 80%, 90%, and 80%, respectively. In situ hybridization showed strong and uniform nuclear positivity of EBV-encoded small RNAs (EBER) in the neoplastic cells of 3 cases.

**Conclusion:**

EBV-positive SCNEC-nasopharynx was exactly rare. The origin of this tumor is still controversial. It may originate from EBV-infected mucosal epithelium like nasopharyngeal carcinoma. Based on our cases and relevant literature, we found EBV-positive SCNEC-nasopharynx as a probably site-specific subtype of SCNEC with differing pathogenetic mechanism. The subtype not only virus positivity but also that it was associated with TILs and did not show p53 or Rb alterations by immunohistochemistry. It may be more responsive to treatment and have a better prognosis than classic SCNEC. We will continue to follow-up these patients and collect additional cases to further understand the unique biology of this rare solid tumor.

## Background

Neuroendocrine neoplasms (NENs) arise in virtually every organ. 70% of them occur in the gastrointestinal pancreas and 25% in the lungs [1]. NENs occurring in the head and neck are relatively rare. The 5th edition of WHO classification of tumors of the head and neck reclassified neuroendocrine tumors into neuroendocrine tumors (NETs) and neuroendocrine carcinomas (NECs). NETs are well-differentiated epithelial NENs and graded as G1 ,G2 and G3 NET based on necrosis, mitoses per 2 mm^2^, Ki67 labeling index. NECs are further subtyped based on cytomorphological characteristics as small cell NECs and large cell NECs. The most common site of NENs (especially small cell carcinoma) in the head and neck is the sinonasal tract. Less common sites of involvement include the larynx, oropharynx, nasopharynx, oral cavity and major salivary glands [1]. Small cell NECs of the nasopharynx (SCNECs-nasopharynx) are extremely infrequent, with only seventeen cases reported in the literature in the past twenty years. The most common cancer in nasopharynx is non-keratinizing undifferentiated carcinoma, which is highly related to Epstein–Barr virus (EBV). EBV-encoded small RNAs (EBER) expression is an important indicator for the differential diagnosis of this type of cancer. It is currently believed that NENs in the nasopharynx are not related to EBV infection. Occasionally, it has been reported in the literature that EBV-positive NENs are mainly large cell NECs [2, 3], while EBV-positive small cell NEC is exactly rare. Herein, we collected 3 cases of EBV-positive SCNECs-nasopharynx in our hospital. Through histological and immunophenotypic analysis, it was found that this solid tumor may have its own unique clinicopathological characteristics. This finding has updated our understanding of EBV-positive SCNECs-nasopharynx.

## Materials and methods

### Case selection

A definite diagnosis of small cell NEC was made based on 5th edition of the World Health Organization Classification of Head and Neck Tumors [1]. Other primary NENs (including Merkel Cell Carcinoma and Ectopic Pituitary Neuroendocrine Tumor), metastatic or secondary small cell NECs were excluded from this study.

Detailed clinical data, such as age, gender, clinical course, symptoms, imaging findings, and treatment details were collected from electronic medical records. Follow-up data were obtained by telephone interviews and/or medical records. The Follow-Up time and the stage were measured from the time of diagnosing SCNEC-nasopharynx.

### Histological assessment

All tissue specimens were fixed with 10% formalin and embedded in paraffin after routine processing. Tissue Sects. (3–4 µm) were stained with hematoxylin and eosin for subsequent microscopic examination. Immunohistochemical analysis and in situ hybridization were performed as part of the standard diagnostic procedures.

### Immunohistochemistry

The antibodies (predilute) and kits were purchased from Maixin Biotech Company (Fuzhou, China) and Zhongshan Biotech Company (Beijing, China), and used according to the manufacturers instructions. The Roch automatic immunohisto- chemistry instrument (BenchMark ULTRA, Ventana, USA) was performed. Sections were counterstained with hematoxylin. All immunostaining were performed as previously described and appropriate positive and negative controls were employed. The primary antibodies that were used in this study were listed in Table 1.

**Table 1:** The primary antibodies used in this study

Antibody	Manufacturer	Species	Clone	Dilution	Stainer
BRG-1	ZSB	rabbit	E8V5B	predilute	ventana
panCK	MXB	mouse	MX005	predilute	ventana
CK 20	MXB	mouse	MX059	predilute	ventana
CD56	MXB	mouse	MXO39	predilute	ventana
CgA	MXB	rabbit	MX018	predilute	ventana
EGFR	MXB	rabbit	EP22	predilute	ventana
INSM-1	ZSB	mouse	A-8	predilute	ventana
INI-1	ZSB	rabbit	OTIR4G9	predilute	ventana
Ki67	MXB	rabbit	MXR002	predilute	ventana
NUT	ZSB	rabbit	C52B1	predilute	ventana
p40	MXB	rabbit	MXR010	predilute	ventana
p63	MXB	mouse	MX013	predilute	ventana
p53	MXB	mouse	MX008	predilute	ventana
Rb	ZSB	mouse	13A10	predilute	ventana
S-100	MXB	mouse	4C4.9	predilute	ventana
Syn	MXB	mouse	MX038	predilute	ventana
TTF-1	MXB	mouse	MX011	predilute	ventana

*Abbreviation*: *MXB *Maixin Biotech Company (Fuzhou, China), *ZSB *Beijing Zhongshan Golden Bridge Biotechnology Company (Beijing, China), *CK *Cytokeratin, *CgA *Chromogranin A, *EGFR *Epidermal growth factor receptor; insulinoma-associated protein 1, *INSM-1 *Syn, synaptophysin

### In situ hybridization

In situ hybridization for the EBV was performed using the inform EBER Probe and automatic immunostainer according to iView Blue Detection Kit recommendations (BenchMark ULTRA, Ventana, USA). ISH protease 3 was used in protein removal and nucleic acid exposures. The developer used Red Counterstain II. For heat-induced epitope retrieval (HIER), the treatment time for cell conditioning (CC2) was varied. A positive reaction was defined as blue staining in the nuclei.

### Literature search

PubMed database was searched for articles published up to April 2024 in any language, using the following keywords: ‘Neuroendocrine carcinoma’, ‘small cell cancer’, ‘small cell neuroendocrine carcinoma’, and ‘nasopharyngeal’. The clinicopathological features of the reported cases, including the present cases, were collected and summarized.

## Results

### Clinical findings

We collected a total of 360 cases of nasopharyngeal neoplasm between July 2019 and April 2024 based on data acquired from the database of the Department of Pathology, Guiqian international general hospital. Among them, three cases met the diagnostic criteria for SCNEC, accounting for 0.8% of nasopharyngeal neoplasms.

**Fig. 1:**
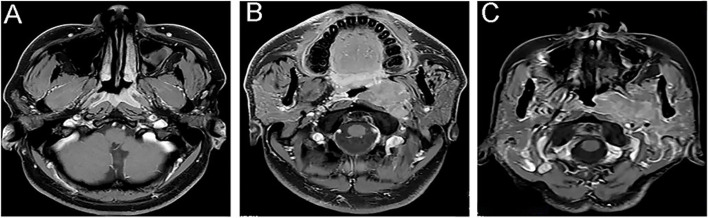
MRI findings of the case 1, 2, 3. MRI showed the thickening area of the roof posterior and bilateral walls of the nasopharyngeal, mainly involved in the left (case 1, **A**). MRI showed a mass involved in the area of roof posterior and left walls, with heterogenous enhancement, and the lesion size was about 35 mm × 44 mm × 65 mm (case 2, **B**). MRI showed the thickening area of the roof posterior and bilateral walls of the nasopharyngeal, mainly involved in the left (case 3, **C**)

Case 1 involved an otherwise healthy 33-year-old male who presented with a left neck mass for 1 month. Nasal endoscopy showed local protrusion on the left side of the posterior wall of the nasopharyngeal. Magnet resonance imaging (MRI) showed thickening of the posterior wall and left upper wall of the nasopharyngeal, and the left parapharyngeal space (Fig. 1A). There were multiple lymph node metastases in areas II and V of the left neck. Systemic examination including careful palpation, abdominal ultrasonography and contrast-enhanced computed tomography (CT) revealed no other tumors. Based on these findings, the tumor was staged as T2N3M0 and the patient received 5 cycles of chemotherapy with docetaxel-cisplatin that was followed by concurrent chemoradiotherapy with triweekly cisplatin and a total dose of 70 Gy delivered in 35 fractions. At the time of this reporting, the patient had been followed for 15 months after initial admission without local recurrence or metastasis to other distant organs.

Case 2 involved a 39-year-old male who admitted to the hospital because of numbness at the corner of his mouth for 3 months, left temporal and facial pain for 2 months, and incomplete closure of the left eye for 6 days. His past history was unremarkable. Nasal endoscopy showed neoplasm was seen on the left side of the nasopharynx, invading the left choana. MRI of the nasopharynx showed (Fig. 1B) space occupying the roof and left wall of the nasopharynx and involvement of surrounding structures. An enlarged lymph node was found on the left side of the neck, suggesting the possibility of metastasis. Abdominal CT showed multiple metastatic tumors in the liver (confirmed on pathological examination). Based on these findings, the tumor was staged as T4N1M1. Subsequently, The patient returned to the local hospital for treatment (not available). At the time of this writing, the patient had been followed for 13 uneventful months after initial diagnosis.

Case 3 involved a 73-year-old female who noticed a gradually growing bilateral neck mass for 1 month. Nasal endoscopy showed a raised neoplasm with surface congestion and ulceration in the nasopharynx.The neoplasm invaded the left choana, full pharyngeal recesses on both sides, and pressured on the pharyngeal opening of the left Eustachian tube. MRI showed (Fig. 1C) that the bilateral walls and parietal posterior wall of the nasopharynx were significantly thickened, and there were multiple lymph node metastases in the bilateral supraclavicular area and neck. General examination, including careful palpation, abdominal ultrasound, and enhanced CT, revealed no additional tumors. Based on these findings, the tumor was staged T3N3M0, and the patient received 4 cycles of immunochemotherapy with Carboplatin-Etoposid-Slulizumab. Followed by 2 cycles of concurrent immunochemotherapy (Carboplatin + Slulizumab) and radiotherapy at a total dose of 50 Gy, divided into 24 doses. At the time of writing this report, the patient had just been completed the last cycle of treatment and was in generally good condition. A follow-up MRI showed that the size of the nasopharyngeal tumor and the original enlarged lymph nodes had significantly reduced.

### Morphological features

**Fig. 2:**
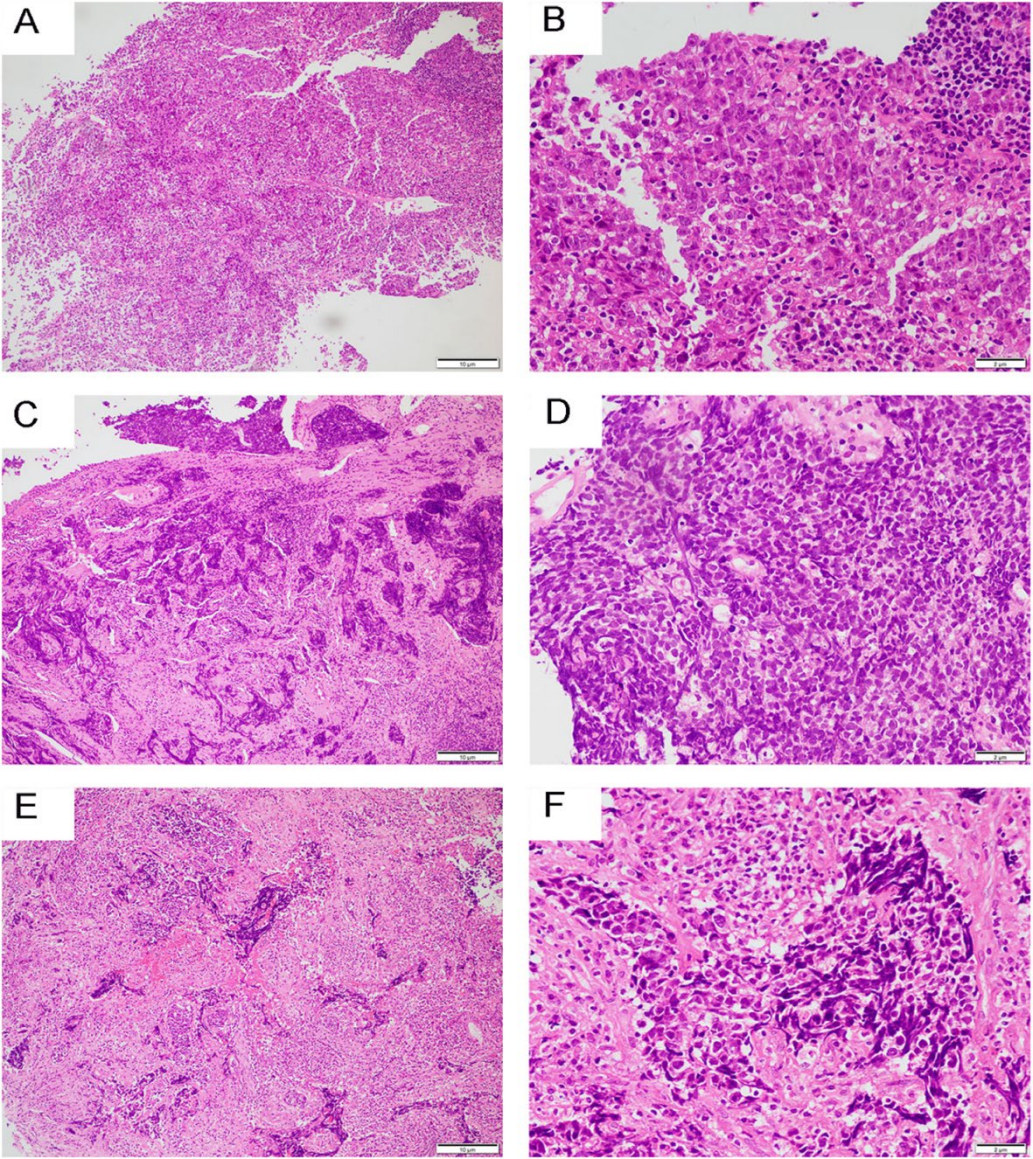
The histologic features of case 1 (**A**, **B**), case 2 (**C**, **D**), and case 3 (**E**, **F**). Magnification A, C, E × 200 and B, D, F × 400

The nasopharyngeal mucosa biopsies were performed in the 3 cases. Their histologic features were similar and showed in Fig. 2, respectively. The surfaces of three cases were covered with squamous or pseudostratified ciliated columnar epithelium which be invasived and ulcerated by tumor cells. The tumor cells located under the surface were smaller than 3 lymphocytes in diameter, scant cytoplasm with hyperchromatic to finely granular or stippled appearing chromatin, inconspicuous nucleoli, nuclear molding and mitotic figures are apparent, > 10 mitoses per 2 mm^2^. They were arranged in the shape of solid nests, sheets, and cords. Patchy or cluster necrosis and apoptotic bodies were easy to observed. Moreover, a large numerous of chronic inflammatory cells were seen in the background all of them.

The tumor located under the mucosa arranged in the shape of solid nests, sheets, and cords, with a large numerous of chronic inflammatory cells were seen in the background all of them (A, C, E). The neoplasm cells were smaller than 3 lymphocytes in diameter, scant cytoplasm with hyperchromatic to finely granular or stippled appearing chromatin, inconspicuous nucleoli, nuclear molding and mitotic figures were apparent (B, D, F).

### Immunohistochemical phenotype and molecular findings

The immunohistochemical findings of the three cases were summarized in Table 2.

**Table 2:** Comparison of the immunohistochemical findings of present cases

Antibody	Case 1	Case 2	Case 3
CK	D +	D +	D +
CK20	-	-	-
CD56	-	-	D +
CgA	-	-	-
Syn	D +	D +	D +
INSM-1	D +	D +	-
EGFR	D +	D +	D +
p53	wild-type	wild-type	wild-type
Rb	retained	retained	retained
p63	-	-	-
p40	-	-	-
S-100	-	-	-
TTF-1	-	-	-
BRG-1	retained	retained	retained
INI-1	retained	retained	retained
NUT	-	-	-
Ki67 (%)	index 80%	index 90%	index 80%

D, diffuse (> 80% of the tumor cells positive reactivity)

**Fig. 3:**
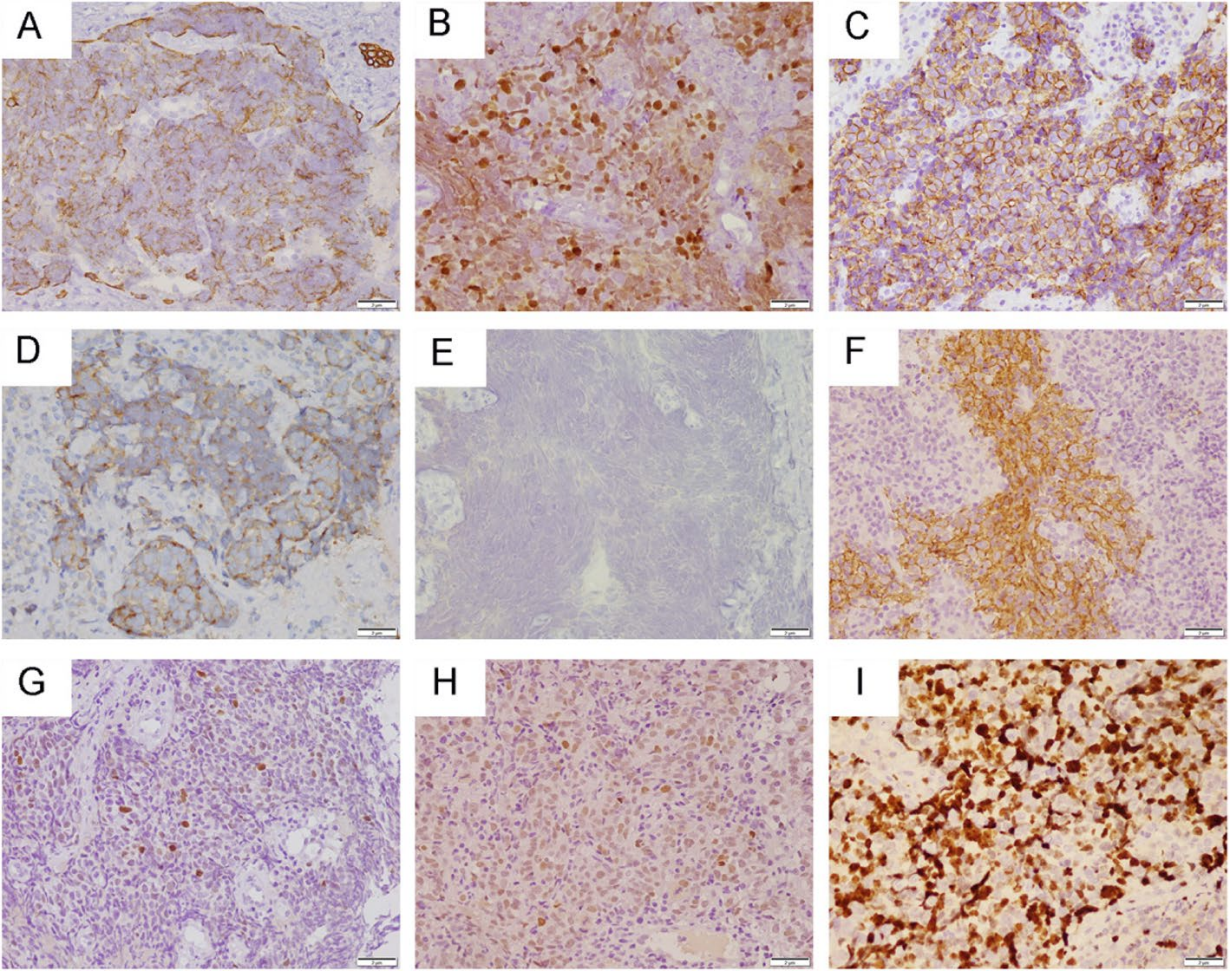
The immunohistochemical features of case 1 (A, D and G), case 2 (B, E and H) and case 3 (C, F and I). Immunostaining for panCK (**A**), INSM-1(**B**), CD56 (**C**), Syn (**D**), p40 (**E**), EGFR (**F**), p53 (**G**), Rb (**H**), Ki67 (**I**). All magnification × 400

**Fig. 4:**
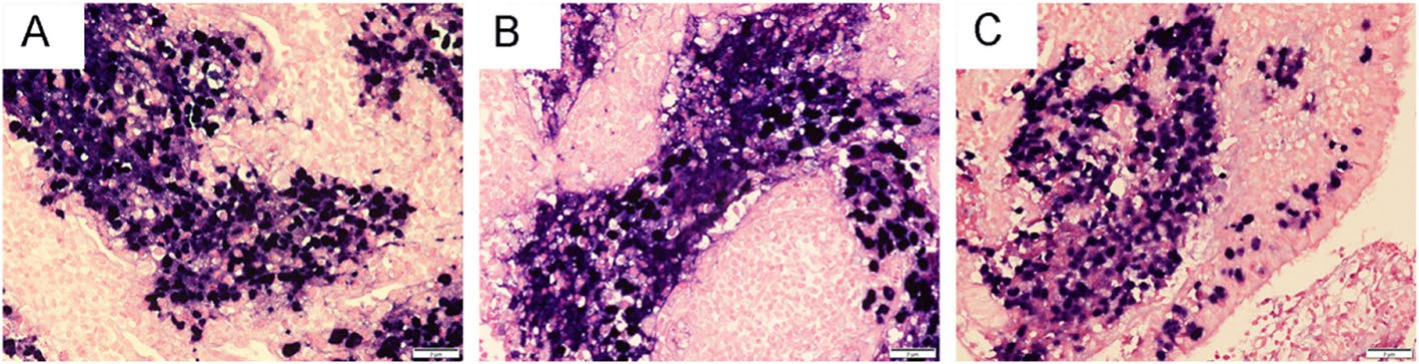
In situ hybridization features of the present cases. In situ hybridization showed strong and uniform nuclear positivity of EBER in the neoplastic cells of case 1 (**A**), case 2 (**B**) and case 3 (**C**). EBV-infected cells in the basal layer of the tumor surface epithelium were observed (C)

All three cases were positive for panCK (Fig. 3A) and EGFR (Fig. 3F). Case 1 and Case 2 diffusely expressed INSM-1 (Fig. 3B) and Syn (Fig. 3D), Case 3 strongly stained for CD56 (Fig. 3C) and Syn. Immunostaining for p40 (Fig. 3E), p63, TTF-1, CK20, S-100 and NUT showed negative. And p53 (Fig. 3G) all showed wild-type expression. BRG-1, INI-1 and Rb (Fig. 3H) were retained. The Ki-67 labeling indices of case 1, 2, and 3 were 80%, 90%, and 80% (Fig. 3I), respectively. In situ hybridization showed strong and uniform nuclear positivity of EBER in the neoplastic cells of present cases (Fig. 4). Case 2 and case 3 (Fig. 4C) were observed EBV-infected cells in the basal layer of surface epithelium and in the immediately adjacent epithelial near the tumor.

### Literature review

The clinicopathology of 20 cases, collected previous literature reports on 17 cases of primary SCNECs-nasopharyngeal [4–18] and 3 cases reported here, were summaried in Table 3. The age of patients diagnosed in these cases ranged from 30 to 80 years, with the most affected age range being 40–60 years. Gender ratio was 12:8 (male vs female), with males advantage. Among them, 11 cases were available for lesion locations by imaging, 10 cases on the lateral and posterior walls of the nasopharynx, and 1 case on the anterior wall. EBV detection information in situ hybridization was provided in 8 cases, and 4 cases (including 3 cases present here) were diffusely positive for EBER. Cisplatin-etoposide + RT was the most commonly scheduled therapy regime. Immunotherapy was added to 2 cases. The longest overall survival time of those reported patients was 4 years, and 8 patients died of the tumor progression.

**Table 3:** Clinicalpathology summary of present cases and historic cases of SCNECs-nasopharynx

NO	Case	Age	Gender	Tumor Site	Stage	EBER	Treatment	Follow-up
1	Current case 1	33y	male	posterior and bilateral walls	T2N3M0	positive	Cisplatin,follow by Aalbumin paclitaxel + Nedaplatin + RT	PR, AWD at 15 months
2	Current case 2	39y	male	posterior and left walls	T4N1M1	positive	NA	PR, AWD at 13 months
3	Current case 3	72y	female	posterior and bilateral walls	T3N3M0	positive	Carboplatin + Etoposid + Slulizumab + RT	PR, AWD at 11 months
4	2007 [4]	43y	male	NA	NA	ND	VP-16 and cisplatin	DOD at 38 months
5	2008 [5]	40y	male	near the sphenopalatine foramen on the lateral wall	NM	ND	Surgery	DOD at 11 months
6	2008 [6]	74y	female	NA	NA	ND	Surgery + chemotherapy	NED at 18 months
7	2009 [7]	66y	female	NA	T4N1	ND	Cytoxan + vincristine lomustine + methotrexate + RT	DOD at 15.2 months
8	2009 [7]	80y	male	NA	T4bN2a	ND	Cisplatin + Etoposide + RT	NED at 5.4 months
9	2011 [8]	41y	female	An irregular left nasopharyngeal mass	T1N1M0	negative	Cisplatin + Etoposide + RT	PR, AWD at 9 months
10	2012 [9]	52y	male	NA	NA	negative	NA	NA
11	2015 [10]	43y	female	right lateral and posterior walls	NA	ND	Cisplatin + Etoposide + RT	PR, AWD at 10 months
12	2015 [11]	54y	male	inferior wall	T3N3M0	ND	Cisplatin + Etoposide + RT	DOD at 32 months
13	2017 [12]	54y	male	extensive nasopharyngeal lesion	T2N0M0	ND	Carboplatin + Etoposid + RT	NED at 4 years
14	2017 [13]	46y	female	extensive mass of nasopharynx	NA	ND	Cisplatin + Etoposide	DOD after third course of chemotherapy
15	2018 [14]	30y	female	posterior wall	NM	ND	ND	DOD before treatment initiation
16	2019 [15]	53y	male	posterior wall	T2N1M0	ND	Chemoradiotherapy	DOD at 16 months
17	2019 [15]	75y	male	posterior wall	T1N0M0	ND	Chemoradiotherapy	NED at 103 months
18	2022 [16]	44y	male	in the left side of the nasopharynx	T4N2M0	negative	Cisplatin + Etoposide + RT	CR at 45 months
19	2023 [17]	59y	female	nasopharynx and left nasal cavity	NA	negative	Cisplatin + Etoposide + RT	CR at 2 years
20	2024 [18]	24y	male	posterior and left walls	T2N2M0	positive	Cisplatin + Etoposide + Carilluzumab + RT	PR, AWD at 2 months

## Discussion

Nasopharyngeal carcinoma (NPC), especially non-keratinizing undifferentiated type (UK-NPC), is known to be highly associated with EBV infection [19], and the most common tumor of the nasopharynx, which rises after the age of 30 years, peaks at 40–60 years. The incidence of SCNECs of the head and neck accounts for only 0.3% of head and neck tumors, 10% of which occur in the nasopharynx [15, 20]. There is currently very little information on SCNEC-nasopharynx, with only 17 cases reported in literature in the past 20 years. Herein, after summarizing a total of 20 cases of SCNEC-nasopharynx reported in this and previous literatures, we found that the most affected age range being again 40–60 years. Gender ratio was 12:8 (male vs female), with still males advantage. Furthermore, clear sites of onsets were provided in 11 cases, 10 of which were located on the lateral and posterior wall of the nasopharynx. And most cases were accompanied by cervical lymph node metastases at the time of diagnosis. These clinical characteristics were very similar to those of NPC, so the diagnosis must rely on pathological examination.

The 3 cases of tumors in this article were all located under the mucosa and had similar morphological characteristics. They had the typical histological features of SCNEC in other organs and extensively expressed neuroendocrine markers, but did not stain squamous cell carcinoma markers p63 and p40. However, it must be noted that many poorly differentiated neoplasms have also been shown to exhibit distinct neuroendocrine differentiation, such as SMARCA4- or SMARCB1-deficient cancers, NUT cancers, melanoma and others. In our study, there was no null expression of BRG-1 and INI-1. NUT and S-100 were negative. Nasopharyngeal SCNEC is not believed to be associated with EBV infection. But the three cases reported here were unexpectedly strong and diffuse positive for EBER, which is an important indicator of NPC. Based on the above histological changes and immunohistochemical phenotype, the diagnosis of EBV-positive SCNECs-nasopharynx were confirmed.

Conventional views ascribe the origin of upper respiratory tract NEN to neuroendocrine cells in the lining epithelia of the organ. The neuroendocrine system of the respiratory passages is composed of specialized innervated endocrine epithelial cells. These cells are located in submucosal gland ducts [6]. These epithelial cells can be seen either as a solitary or clustered neuroepithelial body. Another theory of origin suggests that an altered microenvironment could cause the acquisition “neuroendocrine” phenotypes by “reserve” progenitor [21]. Could the factor leading to changes in the microenvironment be related to EBV infection? EBV is endemic in southern China and Southeast Asia. NPCs occurring in these areas are strong association with it infection. In recent years, studies have shown that EBV infection in NPC occurs in the early stages, and NPC gradually develops from clones of single EBV-infected cells. One of the mechanisms by which EBV infection causes NPC is by regulating signal transduction pathways such as NOTCH to allow nasopharyngeal epithelial cells to have “stem cell-like properties” [19]. NOTCH is an important signaling pathway that controls cell differentiation. Nowadays, this pathway is recognized as being responsible for many human diseases including cancer, where it may function as an oncogene and as a tumor suppressor [22]. Relevant literature indicated that NOTCH is also the main regulator of neuroendocrine differentiation of SCNEC [23]. In the present case 2 and case 3, EBV-infected cells were observed in the basal layer of the tumor surface epithelium and in the immediately adjacent epithelial. This phenomenon was not seen in normal nasopharyngeal mucosal epithelium without the tumor. Therefore, we speculated that these EBV-infected epithelial cells, as previously described, were involved in the pathogenesis of neoplasm and may be the origin of EBV-positive SCNEC- nasopharynx, rather than a concomitant manifestation. If EBV infection also played an important role in this site of SCNEC, it should have similar regional / geographic proclivity to NPC. The four reported cases of EBV-positive SCNEC, all from southern China, appeared to support the above speculation. Of course, the single oncogenic driver model is failing to satisfactorily portray the source of the tumor. However, the following findings also suggested that EBV-positive SCNEC-nasopharynx appeared to be distinct from classic SCNEC: ①A study conducted a comprehensive analysis of somatic genomic changes in SCNEC and found that the complete loss of TP53 and Rb functional genomes in classic SCNEC is obligatory for its pathogenesis [23]. However, these hallmarks of SCNEC were missing in the cases we presented. All 3 cases showed wild-type expression of p53, and retained of Rb.②EGFR is a transmembrane protein with cytoplasmic kinase activity that transduces important growth factor signaling from the extracellular milieu to the cell [24]. It plays an important role in regulating the proliferation, survival, motility, and differentiation of the tumor cells. EGFR is also one of the most important oncogenes. Furthermore, EGFR is necessary for the internalization and fusion of the EBV in NPC cells and perhaps can enhance its survival in the host [24]. It is common expressed in NPC, but rarely in classic SCNEC [24, 25]. Unusually, diffuse expression of EGFR appeared in our three cases like NPC. ③Heavy infiltration of lymphocytes and inflammatory stroma is another common histopathological feature of UK-NPC. However, classic SCNEC are generally defined as “immune-cold” tumors [26]. This is also one of the reasons why immunotherapy for SCNEC is ineffective. However, this phenomenon was observed in all of our cases. The pathological features of this subtype appeared to be more similar to NPC.

SCNEC can arise from multiple anatomic subsites and carries a poor prognosis. Moreover, SCNEC-nasopharyngeal has been reported to have the worst prognosis of all SCNECs of the head and neck [16]. The median OS of patients was 18 months, which is much shorter than that of patients with NPC. The one-, three-, and five-year survival rates of patients who received therapies featuring radiation and chemotherapy were 62.9%, 62.5%, and 56.3%, respectively [16]. Among the cases collected in this article, 12 cases provided accurate clinical staging, and 19 cases provided patient prognosis information. From this data, it can be seen that the lymph node metastasis rate was as high as 83%, and the longest overall survival time of these reported patients was 4 years, 8 patients died from tumor progression. For many years the standard of care for SCNEC has remained unchanged. Cisplatin-etoposide + RT is the most important treatment method. But these treatments have not significantly improved the prognosis of this lethal tumors. In recent years, some new treatment strategies, such as immune checkpoint inhibitors (ICIs) and the application of molecular targets, have been provided the identification of novel potential therapeutic opportunities [27]. Previous literature has shown that SCNEC-nasopharyngeal stage, age (> 70 years), gender, and radiotherapy were significant correlation with overall survival (OS) [16]. Furthermore, the presence of TP53 and Rb mutations are also importantly associated with unfavorable survival outcomes [23]. These two poor prognostic indicators were absent in our cases, and abundant TILs and EBV infection are also associated with good survival in many cancer patients [2, 27]. The patients in this study showed excellent response to chemoradiotherapy. At the time of writing this report, the follow-up times were 11 months, 13 months and 15 months, respectively. Therefore, we surmised that EBV-positive SCNEC-nasopharyngeal may show respond better to treatment and have longer OS than classic SCNEC.

## Conclusion

SCNEC-nasopharynx is a very uncommom tumor. To our knowledge, this is the best comprehensive collection of clinicopathological features of this rare tumor entity. EBV-positive SCNEC-nasopharynx is exactly rare. There are currently only 4 cases reported in the literature (including 3 cases in this article). It is speculated that its origin was related to EBV infection of the nasopharyngeal epithelium. It can be seen from our data that EBV-positive SCNEC-nasopharynx and NPC have similar epidemiological characteristics and incidence sites, furthermore may be different from classic SCNEC in pathogenesis. Based on its characteristic histologic features and immunoprofile, it can be proposed that EBV-positive SCNEC-nasopharynx may be as a site-specific subtype of SCNEC with differing pathogenetic mechanism. This subtype may be more responsive to treatment and have a better prognosis than classic type. We will continue to follow-up these patients and collect additional cases to further understand the unique biology of this rare solid tumor.

## Data Availability

No datasets were generated or analysed during the current study.

## Abbreviations

SCNEC: Small cell neuroendocrine carcinoma
EBV: Epstein–Barr virus
NPC: Nasopharyngeal carcinoma
TILs: Tumor-infiltrating lymphocytes
EGFR: Epidermal growth factor receptor
MXB: Maixin Biotech Company (FuzhouChina)
ZSB: Beijing Zhongshan Golden Bridge Biotechnology Company (Beijing, China)
CK: Cytokeratin
CgA: Chromogranin A
EGFR: Epidermal growth factor receptor
INSM-1: Insulinoma-associated protein 1
Syn: Synaptophysin
